# Tumor Microenvironment in Breast Cancer—Updates on Therapeutic Implications and Pathologic Assessment

**DOI:** 10.3390/cancers13164233

**Published:** 2021-08-23

**Authors:** Joshua J. Li, Julia Y. Tsang, Gary M. Tse

**Affiliations:** Department of Anatomical and Cellular Pathology, Prince of Wales Hospital, The Chinese University of Hong Kong, Ngan Shing Street, Shatin, Hong Kong, China; joshuali@cuhk.edu.hk (J.J.L.); jystsang@cuhk.edu.hk (J.Y.T.)

**Keywords:** breast cancer, tumor microenvironment, stromal response, fibrotic focus, tumor infiltrating lymphocytes

## Abstract

**Simple Summary:**

The tumor microenvironment (TME) in breast cancer plays important roles in tumor behavior and treatment response, making its pathologic assessment critical for disease management. Analysis of the TME is not only limited to research-based technologies but is now incorporated into routine histopathologic reporting for practical clinical application. This review covers the current understanding of the TME of breast cancer, its pathologic assessment relevant for prognostication and treatment strategies, and the cancer therapies that interacts with and/or exploits the TME in breast cancer. As actionable targets are constantly being discovered in the TME, the future approach to breast cancer therapy is likely to combine cancer cell elimination and TME modulation.

**Abstract:**

The tumor microenvironment (TME) in breast cancer comprises local factors, cancer cells, immune cells and stromal cells of the local and distant tissues. The interaction between cancer cells and their microenvironment plays important roles in tumor proliferation, propagation and response to therapies. There is increasing research in exploring and manipulating the non-cancerous components of the TME for breast cancer treatment. As the TME is now increasingly recognized as a treatment target, its pathologic assessment has become a critical component of breast cancer management. The latest WHO classification of tumors of the breast listed stromal response pattern/fibrotic focus as a prognostic factor and includes recommendations on the assessment of tumor infiltrating lymphocytes and PD-1/PD-L1 expression, with therapeutic implications. This review dissects the TME of breast cancer, describes pathologic assessment relevant for prognostication and treatment decision, and details therapeutic options that interacts with and/or exploits the TME in breast cancer.

## 1. Introduction

The understanding of the tumor microenvironment (TME) in breast cancer is rapidly evolving. Experimental findings in the TME are translated to clinical prognostic and therapeutic applications. Traditional grading and typing of breast cancer are based solely on the assessment of tumor cells, but now pathologic assessment of the TME is incorporated in routine breast cancer management. This review covers the components of the TME and their interactions; how the TME reacts to and modulates conventional treatment modalities including radiotherapy and traditional chemotherapy; the laboratory assessment of TME; and the therapeutic agents mediating through changes of TME.

## 2. Components of the TME and Their Interactions

The TME in breast cancer can be classified into cellular, soluble, and physical components [1] (Table 1). The cellular components can be subdivided into local (intratumoral), regional (breast) and metastatic compartments [1,2]. The local compartment refers to the biologic features of the tumor cells and the tumor infiltrating inflammatory cells including lymphocytes, plasma cells, dendritic cells, macrophages and neutrophils [3,4]. The regional compartment refers to the interplay between tumor cells and the adjacent cells in the stroma, particularly at the infiltrating edge, involving stromal fibroblasts, myoepithelial cells, adipocytes and endothelial and vascular/lymphatic endothelial cells [1,5]. The metastatic compartment refers to host cells at lymph nodes and distant organs as metastatic sites, forming new TME [6,7]. Various soluble and physical factors also play a role in tumor progression in the breast and at distant sites, these include enzymes, cytokines and growth factors.

### 2.1. The Local Microenvironment

Although breast cancer is not regarded as an immune hot tumor, a high number of tumor-infiltrating lymphocytes (TILs) can be found in high grade, hormone receptors negative or HER2 positive cancers [8,9,10,11]. TILs in breast cancer are predominately T-cells with much fewer B-cells [12,13]. Different classes of T-cells exert unique effects on the TME. CD8 cytotoxic T-cells eliminate tumor cells by releasing granzyme and perforin, mediated by interferon-γ (IFN-γ) secretion. Among the CD4 T-cells, the type 1 helper (Th1) are induced by IFN-γ and IL-12 signals and license antigen presenting cells for effective CD8 T-cell differentiation and clonal expansion [14,15]. The T helper type 2 (Th2) and type 17 (Th17) cells have more varied roles in breast cancer progression [16,17]. Follicular helper T-cells (Tfh) play important roles in antigen specific B-cell maturation, promoting local memory cell differentiation and supporting the development of tertiary lymphoid organ thus enhancing local anti-tumor immune response [18,19]. Regulatory T-cells (Treg) are important regulators of immune system homeostasis and tolerance, and their presence in TME promotes immunosuppression through immunosuppressive cytokines (interleukin-10 (IL-10), transforming growth factor-β (TGF-β)) and direct cell-cell contact suppression [20]. In general, the presence of Th1 response is associated with better clinical outcome [21]; whereas the Treg can support breast cancer progression [22]. The roles of tumor-infiltrating B-cells remains elusive. They may exert both pro-tumor and anti-tumor effects depending on the composition of the TME and on their phenotypes. Their anti-tumor activities are mediated by the recognition of tumor-specific antigens and antibody production or antigen presenting cell (APC) function [23,24]. B-cells can be found in close proximity with T-cells particularly at tertiary lymphoid structures (TLS) in TME [25], and their presence is considered a favorable prognostic factor [12,25]. Following antigen exposure, B-cells differentiate into plasma cells. They were also found in TLS, reflecting the development of an active anti-tumor humoral response [26]. B-cells can also be a crucial mediator of tumor growth. Regulatory B-cells (Bregs) express inhibitory molecules such as programmed cell death-ligand 1 (PD-L1) and FAS ligands (FasL) along with the production of anti-inflammatory cytokines such as IL-10, TGF-β, and IL-35 that inhibit immune responses [27]. Their presence has been reported in breast cancer tissue and promote breast cancer metastasis by converting resting T-cells to Treg [28] but the clinical significance remains elusive at the moment.

Dendritic cells (DC) are the most potent antigen presenting cells, responsible for presenting antigens, including tumor-derived antigens, to T-cells. Upon interaction with T-cells, DC will become mature and more potent in immune stimulation. Tumor cells inhibit DC maturation; thus tumor infiltrating DC tend to exhibit an immature phenotype with an impaired capacity of tumor-derived antigen cross-presentation and a downregulated display of co-stimulatory molecules [29]. DC are divided into myeloid and plasmacytoid populations with different cell surface protein expression. Myeloid dendritic cells (mDC) mainly act in immune cell activation whereas plasmacytoid dendritic cells (pDC), producing type I IFN, exhibit more tolerogenic properties and demonstrate unfavorable prognostic implications [30]. Tumor associated macrophages (TAMs) are the major innate immune cells in tumor. They exhibit two polarized phenotypes (M1 and M2) depending on cytokine exposure. M1 macrophages, the classically activated phenotype, are stimulated by Th1 cytokines (IFN-γ and tumor necrosis factor (TNF)) and in turn exert anti-tumor effects by producing reactive nitrogen and oxygen species (ROS) and releasing pro-inflammation cytokines [31]. Conversely, M2 macrophages, the alternatively activated phenotype, are activated by Th2 cytokines (IL4, IL10 and IL-13) and are pro-tumor in nature [32]. They suppress anti-tumor response, induce angiogenesis, and support tumor growth and metastasis [31]. Neutrophils are increasingly recognized as tumor infiltrating immune cells. Similar to TAM, tumor associated neutrophils (TAN) exhibit polarized phenotypes. N1 pro-inflammatory and anti-tumor TAN are generated by IFN-γ and IFN-β exposure, whereas N2 anti-inflammatory and pro-tumor TAN are induced by TGF-β exposure [33,34]. TAN can reduce CD8 proliferation and recruit immunosuppressive cells in TME in breast cancer mouse models, but their effects in human patients are unclear [35].

Given the clinical success of immune checkpoint blockade, much attention has been put on immune checkpoint molecules, especially programmed cell death protein 1 (PD-1), and its ligand PD-L1, which is expressed by cancer cells and immune cells (B-cells, T-cells, DC, and macrophages) [36,37]. When engaged by PD-L1, PD1 inhibits T-cell activation and is known to be a major contributor of immune resistance in the TME [38]. PD-1 + TIL are associated with a worse overall survival in breast cancer patients [11,39], and high PD-L1 expression is seen in high TIL, high grade, hormone receptor negative, HER2 overexpressed and triple negative breast cancers (TNBCs) [40]. However, the main clinical relevance of the PD-1/PD-L1 pathway lies not in its prognostic value but in therapies involving PD-1/PD-L1 blockade. CTLA-4, a cell surface receptor found on activated T-cells that induces anergy by competitive inhibition of CD28 receptors, is another example of a targetable immune checkpoint in other human cancers [41].

### 2.2. The Regional Microenvironment

Cancer-associated fibroblasts (CAF) are the most prominent stromal component. Compared to their normal counterparts, CAF display a higher proliferative index and defects in tumor suppressor proteins [42]. Through the effects of soluble factors and matrix altering enzymes such as vascular endothelial growth factor A and TGF-β, and matrix metalloproteinases (MMP), CAF induce tumor progression by promoting angiogenesis, tumor growth and invasion [43]. One of the effects of TGF-β is driving collagen I production, establishing a pro-tumor fibrotic-like microenvironment [44]. Fibrotic foci in breast cancer, now known as an adverse histologic prognostic feature, are now included in the latest World Health Organization (WHO) classification of breast tumors [45]. CAF are a heterogeneous group of tumor stromal cells that are morphologically fibroblast-like. They are not necessarily derived from transformation of normal fibroblasts in the TME, but could origin from different tissues or precursor cells, including stellate cells, bone-marrow-derived fibrocytes, mesenchymal stem cells, or even endothelial cells, adipocytes, pericytes and smooth muscle cells [46]. Immunohistochemically, CAF express α-SMA, FAP, S100A4 and platelet derived growth factor receptor-β (PDGFR-β). However, these markers are not specific, as they can also be expressed in other cell types. Also, each marker may identify different populations of CAF. Using multicolor flow cytometry analysis on Integrinβ1, α-SMA, FSP1, FAP, PDGFR and caveolin1 and single cell RNA sequencing (scRNA-seq), four CAF populations were identified in breast cancers according to differential marker expression. These different phenotypic populations reflected different functionalities and were associated with different breast cancer subtypes. The CAF-S1 subtype was enriched in TNBC and related to immunosuppression [47]. Vascular CAF (vCAF) expressed genes involved in vascular development; the matrix CAF (mCAF) expressed ECM-related genes; and developmental CAF-expressed genes related to stem cells; the latter two may originate from resident fibroblasts and malignant epithelial cells, respectively. The mCAF and vCAF gene profiles can be detected in bulk RNA sequencing data from human patients’ sample with biologic and clinical significance [48]. However, relationship between different CAF subsets has yet to be elucidated, pending further studies on their biomarker expression and/or gene profiling results.

Endothelial cells and adipocytes are other cellular components comprising the breast TME. They also demonstrate mutual and dynamic crosstalk with tumor cells to drive tumor progression. Breast cancer cells are well known to stimulate angiogenesis by vascular endothelial growth factor (VEGF) and TGF-β [49]. Reciprocal angiogenic effects were shown by vascular endothelial cells demonstrating increased VEGF expression, endothelial proliferation, migration and organization when co-cultured with breast cancer cells [50]. Likewise, VEGF-C, which induces lymphangiogenesis in lymphatic endothelial cells, is detected in breast cancers [51]. VEGF-C overexpressing breast cancers are associated with lymphatic vessels invasion, lymph node metastasis and shorter disease-free survival [52,53]. Adipocytes also interact with breast cancer cells. Visceral, but not subcutaneous, adipocytes promote tumor proliferation and induce epithelial-to-mesenchymal transition via IL-6 and IL-8 [54]. The effects of visceral adipocytes on the breast cancer TME are especially pronounced as the breast is composed of abundant fatty tissue [55].

Oxygenation of the regional TME significantly impacts on breast tumor biology [56]. A hypoxic environment induces expression of aggressive phenotype (estrogen receptor negativity), local tumor progression and nodal metastasis [56,57].

Low oxygen pressure and poor perfusion impair the effects of traditional chemotherapy and radiotherapy [57]. An acidic microenvironment confers pro-tumor effects by facilitating an ECM favoring tumor invasion, inhibiting transcellular uptake of chemotherapeutic agents [58]. Overall, hypoxia and acidosis confer an adaptive survival advantage to resistant tumor clones, resulting in clonal selection; they also hamper the cytotoxic effects of neutrophils and lymphocytes by limiting ROS generation, reducing cellular motility and creating a hostile extracellular environment [59].

### 2.3. The Metastatic Microenvironment

As the tumor cells invade into the lymphatics and blood vessels, they initiate a crucial step in the metastasis cascade [60]. High levels of circulating tumor cells (CTC) are associated with an unfavorable prognosis in patients with metastatic breast cancer [61,62]. The regional lymph nodes are frequently the first site of metastasis as CTC may accumulate in the sinus and form lymph node metastases, a key prognostic indicator for recurrence and poor survival. Then the tumor cells may further migrate into distant organs, forming distant metastases [60]. The most frequent sites for breast cancer metastasis are bones and lungs. Cytokines/chemokines and growth factors secreted by tumor cells are believed to induce receptive changes favoring tumor seeding [63,64]. Tumor-containing lymph nodes show altered immune cell composition (higher Treg density) and structure (lymph node enlargement and lymphangiogenesis) compared to native, benign sentinel lymph nodes [6,65,66]. They also show a different cytokine profile and a lower number of CD83 + dendritic cells [67]. Moreover, a substantially lower TIL count, accompanying by an increase in immunosuppressive gene signature was found in breast cancer metastases compared to the primary tumors [68]. The disparity of immune microenvironments among primary lesions and metastatic foci may partly contribute to the poor response to treatment in advanced diseases. Resident cells in metastatic sites could contribute to tumor seeding. In lung metastases, alveolar macrophages preconditioned with breast cancer cells are believed to inhibit dendritic cell by TGF-β [69]. Depletion of alveolar macrophages increased the dendritic cell population and subsequent Th1 IFN-γ production in a murine breast metastatic model [69]. In bone metastases, metastatic tumor cells can activate osteoclasts through direct and paracrine mechanisms to resorb bone, thereby releasing growth and survival factors that enhance tumor progression as well as causing bone damage. Receptor activator of NF-κB ligand (RANKL) and parathyroid hormone-related peptide (PTHrP) by metastatic cancer cells-enhanced osteolysis. Osteoclastic bone resorption, in turn, releases growth factors including TGF-β that stimulate cancer cell proliferation and perpetuate the osteolytic vicious cycle [7,70,71].

## 3. Interaction between the Tumor Microenvironment and Traditional Cancer Therapies

Traditional therapy targets the proliferative advantage of cancer cells over normal cells. Radiotherapy induces double strand DNA breaks, resulting in cell death mainly through mitotic catastrophe and less commonly by apoptosis and cellular senescence [72]. Chemotherapy selectively damages rapidly dividing cancer cells, through generation of DNA adducts (platinoids and alkylating agents) or single- and double-strand DNA breaks (structural analogs of nucleotide precursors and topoisomerase inhibitors). These sustainable damages can lead to apoptosis. Efficacy of traditional therapy can be influenced by the pre-existing inflammatory cells and stromal cells in TME. At the same time, the treatment can modify the components in TME. These changes are manifold and can be pro-tumor or anti-tumor.

### 3.1. The Tumor Microenvironment and Radiotherapy

Radiation as cancer a therapeutic modality has prominent anti-tumor effects. Radiation-induced cancer cell damage/death leads to release of damage-associated molecular patterns (DAMPs), including ATP, high mobility group box 1 (HMGB1), calreticulin and heat shock protein. DAMPs, recognized by immune cells, are potent triggers of inflammatory signals to activate dendritic cells. For example, HMGB1 can activate toll-like receptor 4 on dendritic cells to enhance cross-presentation of tumor antigen and activate subsequent cytotoxic T-cell response [73,74]. This immunogenic cell death (ICD) of cancer cells can change towards a more immunostimulatory TME cytokine profile [75]. Upon irradiation, DNA/micronuclei are released to cytosol and they activate the cyclic GMP-AMP synthase (cGAS)-stimulator of interferon genes (STING) pathway, resulting in the production of type I interferons [76]. The production of IFN can mediate both innate and adaptive immunity through the increase of CD4 and CD8 T lymphocytes and prevent the mobilization of myeloid derived suppressor cells, thereby switching the immune response from immunosuppressive to anti-tumor. Local radiation may contribute to the tumor cell elimination at non-irradiated metastatic sites as locally activated DC can migrate and cross-prime T-cells in lymph nodes to induce a systemic antitumor immunity (the abscopal effect) [77]. In addition, radiation also promotes T-cell infiltration through alteration of chemokine secretion and expression of adhesion molecules. Increased T-cell chemoattractants, such as CXCL9 and CXCL10, indicates an immune active status after RT treatment in breast cancer patients [78]. Radiation also results in upregulation of IL-1β, TNF-α and type I and II interferons, and these induce expression of cell adhesion molecules e.g., VCAM-1 and ICAM-1 on endothelial cells, promoting migration of lymphocytes into the tumor parenchyma [79,80]. The altered gene expression profiles in tumor after radiation can modify neoantigens that are exposed to the immune systems [81]. Other molecules crucial to anti-tumor immunity including MHC class I and MIC A/B, and these are also upregulated by radiation [81].

On the other hand, radiation can also have pro-tumor effects by promoting an immunosuppressive milieu under a chronic inflammatory condition. DAMP-induced chronic inflammation in the TME causes an increase in immunosuppressive populations. For example, after radiation, breast cancer cells produce CCL2 [82], which stimulates TAM recruitment [83] and breast cancer cells to modulate tumor survival and invasion [84]. TAM can further induce angiogenesis and secrete immunosuppressive mediators like IL-10 and TGF-b, which further promote a radioresistant phenotype [85].

Apart from the intrinsic effects on tumor cells, radiation can also act directly on the different immune cells having different radiosensitivity. In animal model, tumor resident T-cells are more resistant to radiotherapy than those found in lymph node [86,87]. Likewise, Tregs, with their activation of PI3K/AKT pathway, are more radioresistant than other T-cell subtypes, and preferentially survive after radiation [88]. These findings corroborated the observation of little changes in number of tumor-infiltrating T-cells after radiotherapy in breast cancer patients [89]. However, in the peripheral circulating lymphocytes compartment, a higher Treg count and lower absolute lymphocyte count, NK-cell count, and B-cell count were seen in breast cancer patients after radiotherapy, and these changes, except the Treg count, were largely normalized after one year [90].

Additionally, radiotherapy can influence the tumor promoting capability of fibroblasts in the TME. Radiation can induce senescence-like fibroblasts that favor tumor growth [91]. Tumor formation can be observed when non-tumorigenic murine mammary epithelial cell line was injected into radiation pre-treated fat pads [92]. CAFs can confer radiotherapy resistance to cancer cells through a paracrine action. High level of CXCL12 expressed by CAF [93] via signaling through its receptor CXCR4 can support tumor cell stemness in breast cancer [94] and contribute to radioresistance. Another key factor for maintenance of breast cancer stem cells, IGF2, is also expressed by breast CAF [95]. Inhibition of IGF2/IGFR1 signaling pathway markedly enhanced radiosensitivity and radiation induced apoptosis of breast cancers [96]. In other human cancers, CAF conferred radioprotective effects to tumor cells through platelet derived growth factor-AA and IGF binding protein-2, - 4 and - 6 (resistance to radiation-induced cell death) and CXCL1 (enhancing DNA damage repair) [97]. The exosomes produced by CAF can activate RIG-1, subsequently transcriptional responses with STAT1 signaling. In parallel, they also activate NOTCH3 on cancer cells. STAT1, via juxtacrine NOTCH3 pathway, mediates treatment resistance in breast cancer [98]. Radiation induces a senescence-like phenotype and MMP production in fibroblasts, leading to extracellular collagen deposition [91]. Lysyl oxidase (LOX) and integrins are also implicated in radiation-induced fibrosis [73,99]. LOX stabilizes extracellular collagen and leads to treatment resistance through changes in local signaling factors and oxygen content [100], whereas β-1 integrin is a mediator radiation resistance in breast cancer cells [101].

**Figure 1:**
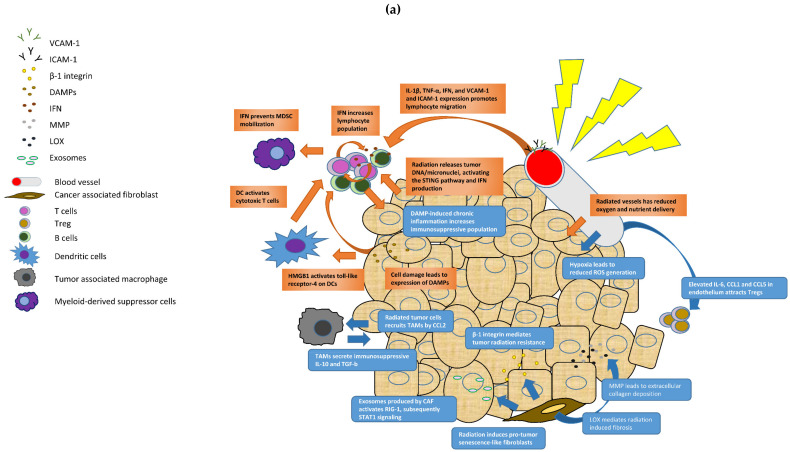

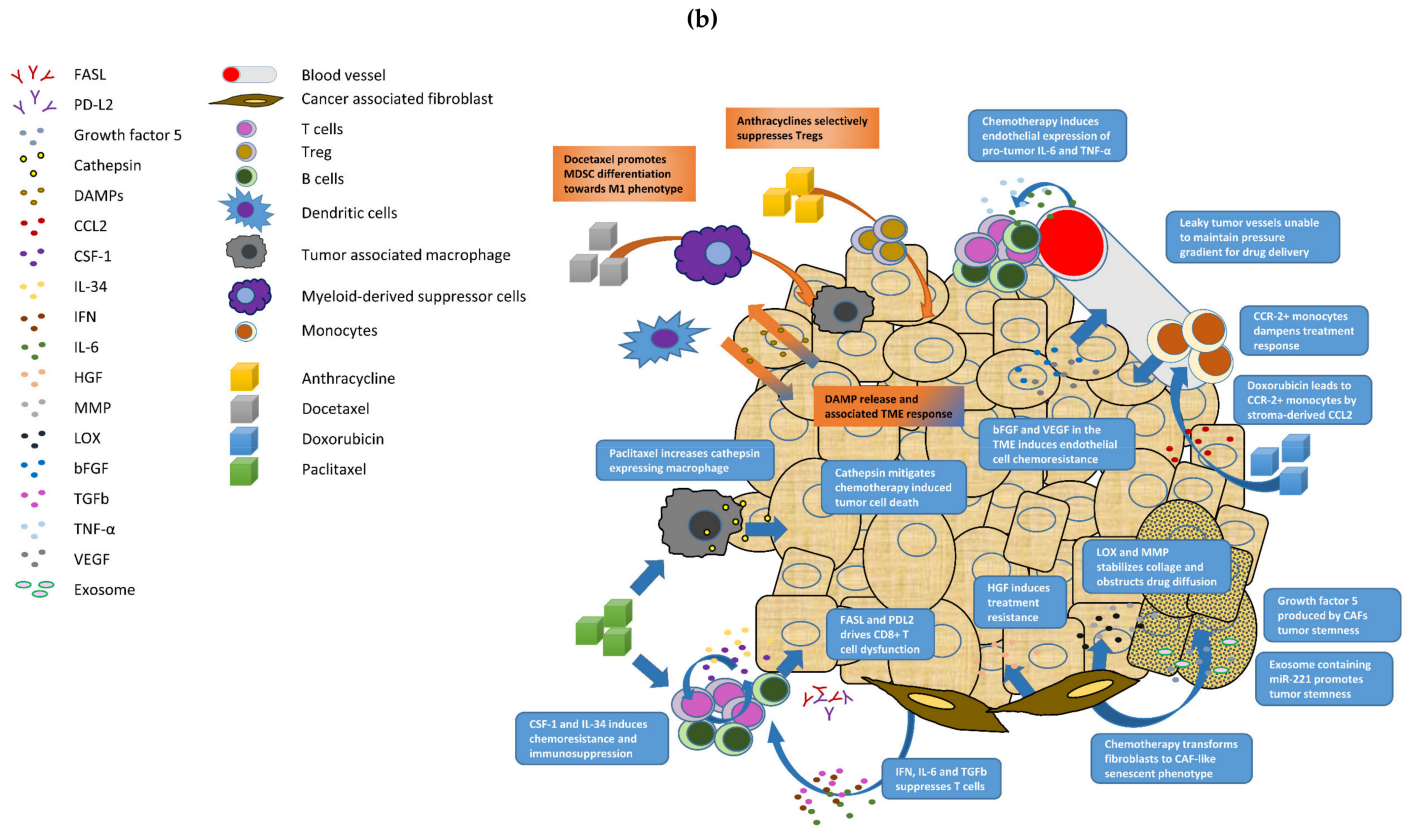
Effects of (**a**) Radiotherapy and (**b**) Chemotherapy on the Breast Cancer Tumor Microenvironment.

Radiation can also show some pro-tumor effects. Radiation-induced damage to tumor vasculature directly reduces tumor oxygenation, drug delivery and nutrients. Although there is theoretical benefit in disrupting nutrient supply to tumor cells [102], radiation actually causes more reduction in therapeutic efficacy and hence jeopardizing prognosis. Tumor-associated endothelial cells in breast cancer are very radiosensitive, even more so than normal endothelial cells [103]. Radiation can increase endothelial cell permeability, detachment and apoptosis [104]. Impaired perfusion leads to tumor hypoxia, reducing ROS generation (oxygen enhancement effect) and ultimately limiting radiation induced tumor apoptosis [57]. Radiation induced DNA damage increases NF-κB activity in endothelial cells, resulting in elevated IL-6, CCL1 and CCL5 production [105]. These cytokines and chemokines attract Tregs and sustain a pro-tumor TME [106,107]. Free radical species resulted from post-irradiation reoxygenation lead to upregulate tumor HIF-1 which induces bFGF and VEGF, thus providing radioprotection for endothelial cells (Figure 1a) [108].

### 3.2. The Tumor Microenvironment and Chemotherapy

Chemotherapeutic agents, similar to radiation, can induce ICD resulting in the release of DAMPs causing activation of subsequent anti-tumor immune response. Common chemotherapeutic agents used in neoadjuvant and primary chemotherapies, such as taxanes, anthracyclines and anti-HER2 monoclonal antibodies can directly induce immunostimulatory effects of tumor cell killing through DC activation [74]. The pre-existing anti-tumor immunity, as indicated by baseline TIL count and CD8 + TIL count, is strongly associated with better response to neoadjuvant chemotherapy (NAC) and better prognosis, especially in TNBCs and HER2 positive breast cancers [9,109]. Indeed, the capacity for a tumor to initiate a robust immune response appears to be predetermined by baseline immune features. Studies monitoring the dynamic immune responses during NAC showed that during treatment, TIL and their immune states were significantly associated with the immune status of pre-NAC samples. Interestingly, a much higher proportion of cases with an immune stimulated state achieved pCR compared to immune cold tumors [110]. In contrast, TIL count was generally lower in post-treatment samples [110,111], probably attributable to lymphodepletion by the chemotherapeutic agents or lessening of anti-tumor immune reaction with the decrease of tumor burden. The post NAC residual tumors demonstrate a more immunosuppressive microenvironment, having decreased fractions of immune stimulatory cell types and increased fraction of M2 TAM [110]. Of note, there could be a dynamic change of immune responses varied by subtypes. The majority of ER + HER2- tumors which were immune cold at baseline remained their cold status on-treatment. On the other hand, TNBCs switch to a more immune-stimulated state or maintained the immune hot status on-treatment. Importantly, this on-treatment response is associated with pCR and predictive to treatment outcome [110]. Chemotherapeutic agents have different effects on different immune compartments. Lymphodepletion is more severe in the CD4 than CD8 T-cell subsets. CD8 T-cells can largely regenerate within a year of treatment but an abnormal bias of memory CD4 T-cells towards inflammatory effectors can persist for years [112]. Anthracyclines selectively suppress Tregs in the TME to elicit ICD [113,114]. MDSC are differentially modulated by paclitaxel and docetaxel. Docetaxel promote MDSC differentiation towards M1 phenotype [115]. Some chemotherapeutic agents may also have minor pro-tumor effects. Paclitaxel increases cathepsin expressing macrophages which can mitigate tumor cell death induced by other chemotherapeutic drugs [116]; they also enhance the expression of colony-stimulating factor 1 and IL-34, resulting in further chemoresistance and immunosuppression [117]. The CCR2-CCL2 axis also lessens response to chemotherapy. In a mouse model of breast cancer, doxorubicin treatment led to recruitment of CCR2-expressing monocytic cells by stroma-derived CCL2, dampens treatment response and promotes tumor re-emergence [118].

Chemotherapeutic agents also act on CAF component in the TME by different mechanisms, and these generally have pro-tumor effects. They can trigger senescence in fibroblasts [119]. Even in the absence of tumor cells, chemotherapeutic agents can transform fibroblasts into a CAF-like senescent phenotype which produce pro-tumor inflammatory cytokines, including IFN and IL-6 [119]. CAF can also produce growth factors and exosomes that can modulate drug responses. CAF via its fibroblast growth factor 5 expression and production of fibrillary collagen are capable of inducing and maintaining a stem-like phenotype in TNBC cells in vivo [120]. HGF secreted by CAF signaled Met phosphorylation in breast cancer cell to induce treatment resistance [121]. Exosome-containing CAF derived miR-221 also promotes the development of cancer stem cell phenotype and triggers the evolution of therapy resistant metastases [122]. Delivery and efficacy of chemotherapeutic agents is dependent on the ECM composition (stiffness) of the tumor microenvironment. CAF remodel the ECM through enzymes such as LOX and MMP [99,123,124], stabilizing collagen and forming a rigid and dense ECM obstructing diffusion of chemotherapeutic agents and oxygen [123,124,125]. CAFs can also influence the immune components of TME and promote an immunosuppressive environment. IL-6 and TGFb produced by CAF have well established role in T-cell suppression [126]. Animal studies showed that CAF can drive dysfunction of CD8 T-cells in an antigen dependent manner via PD-L2 and FASL by antigen cross-presentation [127]. In clinical breast cancers, an inverse association of CAF and CD8 T-cells can be demonstrated [47].

Blood vessels in the TME are also affected by chemotherapeutic agents. In breast cancers, the blood vessels are lined by defective endothelial cells, forming vessels with irregular diameters and aberrant branching patterns [128]. These tumor vessels are leaky (abnormally large pore size), and blood flow is reduced [128,129]. A decreased blood flow impairs chemotherapy delivery and tissue oxygenation. Leaky tumor vessels are unable to maintain a pressure gradient across the intravascular and extravascular compartments, resulting in peritumor edema thus further limiting transvascular distribution of chemotherapeutic agents [129]. Chemotherapy triggers expression of IL-6 and TNF-α in endothelial cells [105,130], fostering a pro-tumor inflammatory microenvironment (IL-6) and conferring chemoresistance to breast cancer cells (TNF-α) [130]; at the same time, endothelial cells gain chemoresistance by exposure to bFGF and VEGF present in the TME (Figure 1b) [131,132].

## 4. Pathologic Assessment of the Tumor Microenvironment

Until recently, the epicenter of pathologic assessment of breast cancer has the tumor cells. Clinical staging, histologic grading, biomarker assessment, and gene expression profiling were the mainstay of prognostic factors guiding treatment decisions. Further insight into the underlying mechanisms of the breast TME resulted in the refinements of routine pathologic reporting, in particular the inclusion of TILs and fibrotic foci in the latest WHO classification of breast tumors as a prognostic parameter. Recent advances in techniques and parameters for breast cancer TME assessment are described (Table 2).

### 4.1. Histologic Assessment of the Tumor Microenvironment

**Figure 2:**
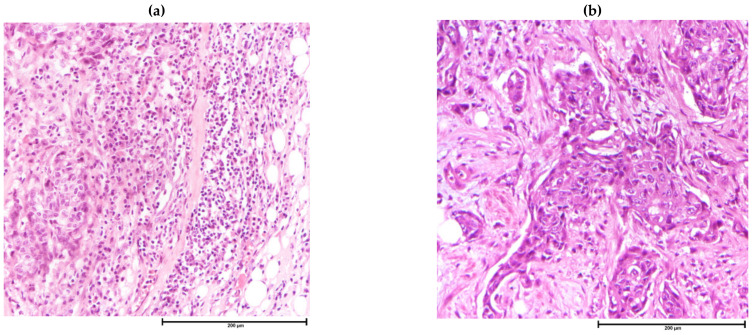
Tumor infiltrating lymphocytes on H&E sections. (**a**) Breast cancer with dense stromal infiltrating lymphocytes, H&E, 200× magnification; (**b**) Breast cancer with sparse lymphocytic infiltrates, H&E, 200×.

Assessment of TIL is recommended to be performed on H&E-stained tissue sections under light microscopy with high-power magnification and reported as percentage area occupied by mononuclear inflammatory cells, including lymphocytes and plasma cells, in the total stromal area within the tumor border (Figure 2) [3]. 

**Figure 3:**
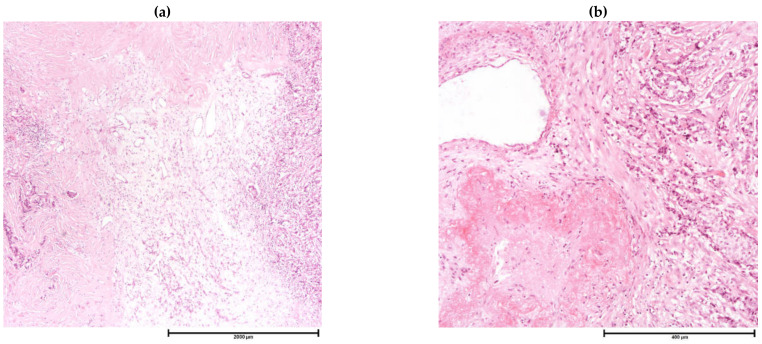
Fibrotic Foci on H&E Sections. (**a**) H&E, 20×; (**b**) H&E, 100×.

Despite being a relatively crude method of assessment and incapable of differentiating the lineage of lymphocytes nor subtyping T-cells, there is strong evidence for recommending histologic scoring of TIL in breast cancer [3]. Although a high TIL level in breast cancers is associated with unfavorable subtypes [8,9,10,11], there is a positive association with a good prognosis and better response to NAC, particularly in TNBCs and HER2 + breast cancers [9,109,133,134]. Recently, attention has also been paid on the clinical value of spatial arrangement of TIL in breast cancer. Applying computational and deep learning techniques on whole slide scanned images, specific TIL clustering patterns showed prognostic implications [135,136]. Lymphoid clustering in form of tertiary lymphoid structures (TLS) (which contain high endothelial venules (HEV) with T-cell and B-cell zones resemble secondary lymphoid organ) is indicative of an active immune response. The presence of TLS is associated with mostly with favorable prognosis in breast cancer. Importantly, compared to TIL, the presence of TLS provided additional significant prognostic information [137,138]. TLS cannot be reliably identified based on H&E-stained slides and requires immunohistochemically stained tissue for its associated components, e.g., B-cell zone surrounded by T-cells, follicular T-cells and HEV. Identification and quantification of specific immune cell types require ancillary techniques and will be discussed in the following sections. Fibrotic focus was initially described as a distinct area of fibrosis within a tumor [139]. When present, it is associated tumor aggressiveness and poor clinical outcome independent of other prognostic variables [139,140]. In more recent trials, presence of central necrosis and/or fibrosis in breast cancers correlated with a shorter DFS [141]. One should note that morphologically FF can vary from highly cellular fibroblastic proliferation to marked hyalinization. Currently, any reactive fibrotic area measuring at least 1 mm within the tumor, with or without necrosis, is considered as a fibrotic focus (Figure 3) [45]. However, the heterogeneity within FF has not been taken into account.

Histologic evaluation of TME also include assessment on vessel density and invasion. Intratumoral microvessel density (MVD) reflects tumor angiogenesis and predicts poor survival in breast cancer [142]. Accurate and reproducible measurement on light microscopy is experience-dependent and necessitates the use of immunohistochemistry (IHC) [143]. High MVD, vascular and lymphatic, are associated with lymph node metastasis and survival, in both treatment naïve and post-NAC tumors [5,142]. Such observations however were unsubstantiated in node-positive breast cancers. Lymphovascular invasion of tumor into peritumoral vessels suggests tumor intravasation and propagation and is associated with a higher risk of recurrence and a shorter survival in early-stage breast cancers [144,145].

### 4.2. Concomitant Histologic Changes in Non-Tumorous Tissue

The effects of radiotherapy and chemotherapy on the TME can be identified on post-NAC breast resection specimens. These changes include alterations in TILs levels [146], and stromal changes such as fibrosis and hyalinization [147,148]. In the axillary lymph nodes, the normal lymphocyte population can be depleted, and replaced by fibrotic tissue with macrophages [148]. The histologic effects of radiation are less well described, as radiotherapy is usually given in the adjuvant or palliative settings. However, post radiation fibrosis is a common clinical complication in breast cancer treatment [149], and autopsy studies demonstrate tissue fibrosis in the irradiated fields in breast cancer patients [150]. Microvessels can display telangiectasia, or rupture resulting in permanent loss after irradiation [151], whereas medium-sized vessels undergo intimal macrophage aggregation, myointimal proliferation, medial hyalinization and fibrinoid necrosis [151].

Soluble factors present in the TME are believed to enter the circulation and induce pre-metastatic changes at distant sites [64]. Increased lymphatic vessel volume were seen downstream to invasive breast cancers in animal models [152]. Lymphangiogenesis preceded metastasis in melanoma [153]. Other recognizable histologic reactive changes in draining lymph nodes are fibrosis, sinusoidal hyperplasia and follicular hyperplasia [154,155]

### 4.3. Immunohistochemistry

#### 4.3.1. Tumor Infiltrating Lymphocytes and Tumor Associated Macrophages

**Figure 4:**
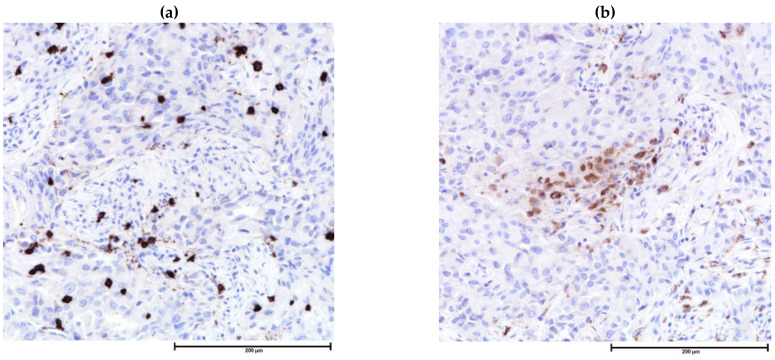
Immunohistochemistry for immune cells in the tumor microenvironment. (**a**) CD8 stain highlights cytotoxic T-cells among lymphocytes, CD8, 200×; (**b**) CD68 highlights tumor associated macrophages which are difficult to identify by H&E, CD68, 200×.

Differentiation of helper, cytotoxic, regulatory T-cells and B-cells on H&E-stained sections by morphology alone is impossible. Through IHC, there is direct visualization of cell surface proteins on light microscopy while preserving tumor and stroma morphology. CD3 (pan T-cell), CD4 (helper T-cell), CD8 (cytotoxic T-cell), CD25/FOXP3 (Treg), CXCL13 (follicular helper T-cell), CD56 (NK cell), CD20 (B-cell) and CD138 (plasma cell) are markers used for subtyping TILs [156] and are available in most hospital laboratories. IHC assessment and prognostic implications of T-cell subsets-cytotoxic, [133,157,158], helper and regulatory [133,157,158,159], B-cells [160,161,162], plasma cells [163] and macrophages [8,164,165] (Figure 4) are summarized in Table 2. Dual-stain IHC, immunofluorescence, multiplex, computer-assisted analysis, automated counting and/or a combination of the above techniques are well-developed on research-based platforms [166], and studies on TILs of breast cancer yielded results consistent with classical single chromogen-based IHC [167,168] studies. IHC and machine scoring, if and when validated, can provide a more robust, reproducible and easy option for routine assessment of TME in routine clinical practice [3].

#### 4.3.2. Programmed Cell Death-Ligand 1

Recent clinical trials have led to FDA approval of immune checkpoint inhibitors (atezolizumab and pembrolizumab) for locally advanced or metastatic breast cancers [169]. PD-L1 testing by IHC is the required accompanied diagnostic test [170]. Initial phase I (KEYNOTE-028) [171] and phase II (KEYNOTE-086) [172] with pembrolizumab monotherapy demonstrated durable response in naïve PD-L1 positive and a subset of previously treated metastatic TNBC [172], as well as modest response in hormone positive/HER2 negative breast cancers [171]. However, the more recent KEYNOTE-119 trial did not demonstrate survival benefit comparing pembrolizumab versus chemotherapy, in previously treated metastatic TNBCs [173]. Trials involving combination therapy showed more encouraging results. The addition of atezolizumab to nab-paclitaxel prolonged progression-free survival in both PD-L1 positive and, at a lesser but still significant degree, PD-L1 negative metastatic TNBC [174]. Similar observations were also found with pembrolizumab with chemotherapy for metastatic TNBCs and early TNBCs. Compared to chemotherapy alone, a significant improvement in PFS, particularly for those with PD-L1 expression, was found in the KEYNOTE-355 for untreated locally recurrent or metastatic TNBCs [175]. For early TNBCs (KEYNOTE-522), the percentage with a pCR was significantly higher among those who received pembrolizumab plus platinum-containing NAC than those with NAC alone [176].

**Figure 5:**
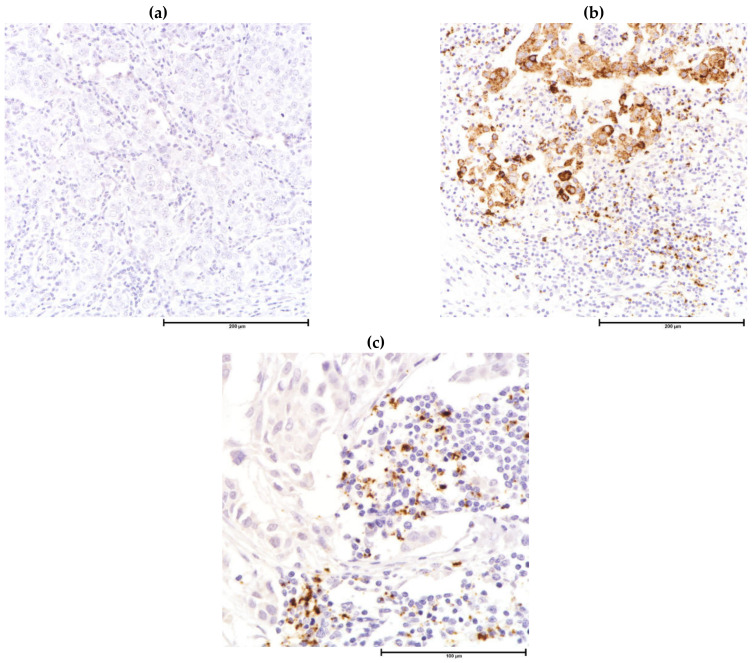
PD-L1 Immunohistochemistry. (**a**) Example of negative PD-L1 expression in tumor and immune cells, Ventana SP142, 200×; (**b**) Positive PD-L1 staining in tumor and immune cells, Ventana SP142, 200×; (**c**) Positive staining in immune cells only, Ventana SP142, 400×.

In all these trials, PD-L1 expression correlated, to certain extent, with efficacy of PD-1/PD-L1 inhibitors. However, these IHC assays for PD-L1 are developed independently for each drug with different antibody clones, staining protocols and platforms, assessment on tumor cells and/or infiltrating immune cells and cutoffs [170]. Clone 22C3 (Dako) and SP142 (Ventana) are companion assays for pembrolizumab and atezolizumab respectively. The Dako PD-L1 IHC 22C3 pharmDx assay defined PD-L1 positivity as a combined positive score of ≥1, while in the Ventana PD-L1 IHC SP142 assay, positivity was defined as ≥1 % expression in tumor-infiltrating immune cells (Figure 5) (Table A1). The different assays have been specifically implemented in relation to the clinical evidence from trials and validated with specific platforms [177,178,179]. Regardless, in addition to specific companion diagnostics, 22C3 (Dako) and SP263 (Ventana) are antibody clones generally accepted by pharmaceuticals for clinical use. 

#### 4.3.3. Dendritic Cell Markers

Among the two different types of DC, pDC appear as round to oval cells with an eccentric nucleus and prominent endoplasmic reticulum and Golgi apparatus (plasmacytoid appearance), whereas mDC are stellate [180,181]. Quantitative assessment of DC is impractical by morphology alone. Identification and typing of dendritic cells require IHC. pDC exhibit a CD123+/CD45RA+/CD11c−/CD13−/CD33−/CD83− phenotype, as opposed to a CD11c+/CD13+/CD33+/CD123−/CD45RA− phenotype in mDC [180,181,182]. pDC are also negative to S100 protein [183]. High levels of both DC types are accompanied by increased T-cell infiltration [30,184]. Some studies demonstrate better prognosis in breast cancer patients with high DC infiltration [30,185] while others were only able to demonstrate associations between mDC and tumor characteristics, such as grading [184,186]. Whether or not DC infiltration demonstrates independent prognostic value of TILs remains unclear. Studies using CD68 (a macrophage marker) for assessment of TAM found negative correlation with survival [187,188], but paradoxically found lower CD68 + TAM in node positive patients [189,190]. Attempts to refine TAM assessment by using CD206, an M2 macrophage marker), did not show prognostic significance [191]. The evidence on the prognostic value of quantifying dendritic cells and macrophages in clinical setting remains inconclusive.

#### 4.3.4. Other Markers

**Figure 6:**
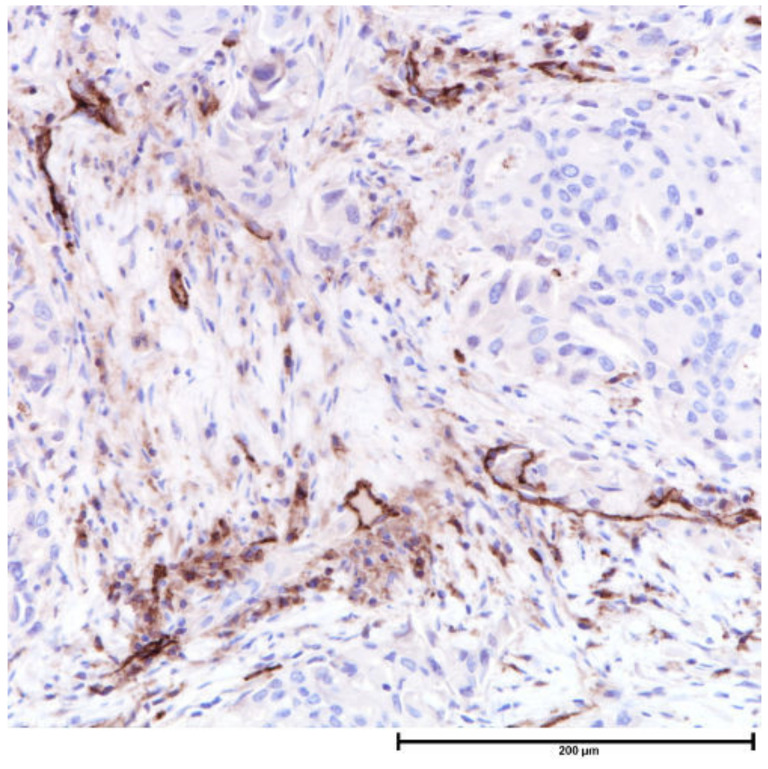
CD31 stain for assessment of microvessel density, CD31, 200×.

CD31 and D2-40 are vascular and lymphatic markers respectively [5] (Figure 6). These markers are useful to assess MVD as histologic assessment can be inaccurate [143], due to the morphologic overlap between vascular and lymphatic channels, and the difficulty in identifying small collapsed vascular lumens histologically. Intratumoral and stromal VEGF expressions correlated with microvascular density [5]. A high microvascular density at the primary tumor is retained at metastasis in lymph nodes [192].

### 4.4. Molecular Methods

Multigene assays like Oncotype DX and Mammaprint have been used for guiding chemotherapy in hormonal receptor positive HER2 negative breast cancer patients. They relied mainly on genes related to tumor intrinsic properties, including tumor proliferation, invasion and signaling pathway. There is accumulating evidence supporting the clinical significance of stromal and immune gene signatures as prognostic or predictive markers for breast cancer. A 26-gene derived from the tumor stroma reflected different clinical outcome in breast cancer. The good outcome cluster overexpressed genes representing Th1 response while the poor outcome cluster showed markers of an increased hypoxia and angiogenic response [193]. Another study focused on a stromal metagene, composed by the expression of 50 genes with a prominent exhibition of ECM proteins. The signature predicted resistance to both anthracyclines and a taxane-based regimen in ER-breast cancers [194]. In line with the prognostic and predictive value of high TIL in ER-cancer, immune gene signatures or the expression of immune-related genes have been reported to be associated with outcomes in ER-cancer subsets [195,196]. Many of these involved genes are related to elevated T/B-cell functions [197]. Immune gene signatures may be useful to select patients who are mostly like to benefit from NAC or adjuvant radiotherapy in breast cancer. One study showed a higher gene expression of cytotoxic molecules, T-cell receptor signaling pathway components, cytokines related to Th1 and Tfh cells, and B-cell markers is correlated with pCR in TNBC patients treated with NAC [198]. Another study developed radiosensitivity and immune gene signatures for predicting benefit of radiotherapy and showed combining these signatures further improved patient stratification in response to adjuvant radiotherapy [199].

Recent advances in single cell RNA sequencing (scRNA-seq) have provided a powerful tool to reveal the complexity of TME and its relationship to breast cancer treatment/outcome. As mentioned, subclasses of spatially and functionally distinct subclasses of CAF have been identified through scRNA-seq. Among them, vascular CAF and matrix CAF signatures have prognostic power [48]. Of note a mCAF signature was also correlated to the previously reported treatment-predictive stromal signature [194]. For CD45 + immune cells, scRNA-seq revealed significantly increased heterogeneity of intratumoral cells of both lymphoid and myeloid cell lineages. There was a markedly expanded phenotypic space in response to environmental stimuli in breast cancer tissue in comparison to normal breast tissue [200]. These observations will contribute to a better understanding of the underlying mechanisms by which TME promotes and resists tumor progression. The scRNA-seq study focused on breast cancer T-cells found a tissue-resident memory subset associated with improved prognosis survival in early-stage TNBCs and provided better prognostication than CD8 expression alone [201]. For therapeutic response, in-depth analysis mainly focused on breast cancer cells at single cell level for their genetic and phenotypic characteristics [202,203]. For analysis on TME, only one pre-clinical study using scRNA-seq data showed that heme oxygenase-1 (HO-1) derived from the myeloid lineage affect the efficacy of immunostimulatory chemotherapy [204]. The refinement of analysis at the single cell level may be crucial for further understanding of TME. Spatially mapped expression data may provide more information on the cellular organization and cell-to cell interactions in tumor and its TME. mRNA in situ hybridization is another approach for analyzing expression of selected genes in specific TME components [187].

## 5. Interplay between the Tumor Microenvironment and Cancer Therapies

### 5.1. Therapies Targeting the Tumor Microenvironment

The PD-1/PD-L1 inhibitors represent a major development in drugs directly acting on the TME in breast cancer. As discussed earlier, PD-1/PD-L1 inhibitors restores cytotoxic T-cell response to cancer cells and is approved in the treatment of metastatic breast cancer [169]. Cytotoxic T-lymphocyte antigen-4 (CTLA-4) is another immune checkpoint under active investigation [205]. CTLA-4 inhibitors include mainly tremelimumab and ipilimumab, which have been approved for other tumors, such as melanoma, non-small cell lung cancer and advanced kidney cancer. In breast cancers, CTLA-4 inhibitors are mainly tested in combination with other therapeutic treatments, including endocrine therapy and anti-PD-1/PD-L1 inhibitors. Findings suggested CTLA-4 inhibition can increase immune activities in patients, but its clinical benefit remains to be demonstrated [206]. Many emerging immune checkpoints targets, e.g., lymphocyte activation gene-3 (LAG-3), T-cell immunoglobulin and mucin domain-containing protein 3 (TIM-3), T-cell immunoglobulin and ITIM domain (TIGIT) and B7-H3, are either in the clinical trial or under active development in breast cancer [205]. Other immunotherapeutic strategies are also under clinical investigations [207]. One strategy is adoptive T-cell therapy using chimeric antigen receptor (CAR-T). CAR-T-cell therapy has been successfully developed as treatment for hematologic cancers. Their use in breast cancer has been challenged by the limited number of known breast tumor neoantigens that can be targeted and the immunosuppressive TME. Despite that, some potential neoantigen targets are being tested in phase II trials. Chimeric CAR-T-cells targeting both antigenic target and other TME cells are also being developed to overcome the immunosuppressive TME [208].

Apart from immune cells, CAF represent another potential therapeutic target. Strategies for CAF-related therapy included targeting the ECM components, ECM remodeling enzymes and targeting CAF via its surface markers/signaling pathways. ECM proteins provided structural signals and support for tumor cells. Hyaluronan (HA), a large glycosaminoglycan produced by CAF, is a key contributing ECM component in stromal fibrosis and tumor progression [209]. A pegylated recombinant human hyaluronidase has been developed to degrade intratumoral ECM and is in early clinical studies [210]. In addition, the treatment efficacy in targeting ECM remodeling enzymes (MMP and LOX inhibitors) [211,212] is supported by strong pre-clinical data. Another CAF-targeting approach is depleting CAF via its surface biomarker. Antibodies against FAP, a major CAF biomarker, have been developed [213]. Clinical trials targeting CAF have been mainly conducted in pancreatic cancers and colorectal cancers; these trials have yet to demonstrate clinical success. Current direction is combining CAF targeting approach with immunotherapeutic treatments [214]. Further developments are required before these approaches can be applied to breast cancer patients.

Additional focus on TME targeting is on overcoming tumor hypoxia and restoring chemo- and radiosensitivity by hyperbaric oxygen and oxygen therapeutics. So far, the results were still discouraging, but improved novel agents are currently under clinical trial [215]. Regarding angiogenic inhibitor, the modest effects of Bevacizumab, a VEGF inhibitor, and reports of serious side-effects led to the removal of FDA-approval in breast cancer [216,217]. For bone metastasis, denosumab, a monoclonal antibody against RANKL inhibiting osteoclast function, showed a superior effect than bisphosphonates in delaying/preventing skeletal related events in a phase III randomized clinical trial [218] (Table A2).

### 5.2. Interaction between the Tumor Microenvironment and Traditional Therapies

Hormonal therapy and HER2 inhibitors exert indirect effects on the TME in addition to their direct effects in tumor cells. Hormone receptors are involved in regulation of many immune cells in the TME [219]. Hormonal inhibition decreased tumor infiltrating Tregs [220] and increased circulating NK-cell activity [221]. Trastuzumab treatment down-regulates CCL2 production in animal experiments [222], and increases PD-1 expression, total immune cell and follicular helper T-cell infiltration clinically in HER2-enriched breast cancer [223]. The immune cell composition of the TME influences trastuzumab efficacy [223,224]. Traditional chemotherapeutic agents interact with immune cells in the TME as well [113,114,116,146,225]. However, the effects of these traditional breast cancer therapies on the TME are mainly collateral.

## 6. Conclusions

Understanding TME in breast cancers is important in research, development and clinical management. Analysis of the TME is not limited to state-of-the-art technologies but is now incorporated into routine histopathologic reporting. Actionable targets are constantly being discovered in the tumor infiltrating immune cells, stromal cells, and the metastatic microenvironment. The future approach to breast cancer therapy is likely to combine cancer cell elimination and TME modulation, hopefully achieving an enhanced synergistic effect.

## Figures and Tables

**Table 1:** Components of the tumor microenvironment with delineated functions.

Component	Local	Regional	Metastatic
Cellular components	Tumor cells Lymphocytes T-cells Helper T-cells Cytotoxic T-cells Regulatory T-cells Follicular helper T-cells B-cells Plasma cells Macrophages Neutrophils Dendritic cells Plasmacytoid dendritic cells Myeloid dendritic cells	Fibroblasts Adipocytes Myoepithelial cells Endothelial cells Capillaries Lymphatics	Lymph node Immune cells Lymphatics Blood Peripheral immune cells Distant organs Bone (osteoclasts) Lung (alveolar macrophages)
Soluble factors	Matrix remodeling enzymes Lysyl oxidase Matrix metalloproteinase Cytokines Interferon-γ Interleukins Tumor necrosis factor Macrophage colony-stimulating factor Growth factors Transforming growth factor-β Vascular endothelial growth factor		
Others	pH Oxygen levels		

**Table 2:** Prognostic pathological parameters.

Parameter	Prognostic implication	Assessment	Significance
Histology			
Tumor infiltrating lymphocytes *	Favorable	Percentage area occupied by mononuclear inflammatory cells divided by total stromal area at the tumor border	Predicts survival and response to neoadjuvant and adjuvant treatment in TNBCs and HER2 + breast cancers
Fibrotic foci *	Unfavorable	Reactive fibrotic area measuring > 1 mm within the tumor, with or without necrosis	Independent factor for shorter disease-free survival
Lymphovascular invasion *	Unfavorable	Tumor permeation into peritumoral vessels	Risk factor for recurrence and shortened survival in early-stage breast cancers
Immunohistochemistry			
Helper T-cells	Uncertain	CD4	Associated with better pathological response but also aggressive tumor features
Cytotoxic T-cells	Favorable	CD8, TIA-1, granzyme	Predicts survival and response to adjuvant treatment
Regulatory T-cells	Uncertain	CD4, CD25, FOXP3	Failed to demonstrate association in survival
Follicular helper T-cells	Favorable	CD4, CXCL13	Associated with pathological complete response to neoadjuvant chemotherapy
B-cells	Favorable	CD19, CD20, CD79a, PAX5	Better treatment response and survival in high-grade breast cancers
Plasma cells	Likely unfavorable	CD38, CD138	Limited data suggests decreased survival
PD-L1 *^, #^	Indication for PD-L1 inhibitor	PD-L1 expression on tumor cells and/or infiltrating immune cells, dependent on antibody clone	Indication for PD-1/PD-L1 inhibitors
Plasmacytoid dendritic cells	Likely favorable	CD123, CD45RA	Associated with increased T-cell infiltration, variably associated with prognosis and tumor grading
Myeloid dendritic cells	Likely favorable	CD11c, CD13, CD33	
Macrophage	Unfavorable	CD68	Unfavorable prognostic factor for survival
Microvessels (capillaries)	Unfavorable	CD31, CD34	Poor prognostic factor for node-negative breast cancers
Microvessels (lymphatics)	Unfavorable	D2-40 ± CD34	

* Recommended as part of standard reporting; ^#^ applies to metastatic disease only.

## Data Availability

Data sharing not applicable to this article as no datasets were generated or analyzed during the current study.

## Appendix A

**Table A1:** Scoring methods for PD-L1 IHC.

Scoring Method	Interpretation
Combined positive score	Number of PD-L1 positive tumor cells, lymphocytes and macrophages divided by total number of tumor cells x 100
Tumor proportion score	Number of PD-L1 positive tumor cells divided by total number of tumor cells x 100
Tumor infiltrating immune cell (IC) score	Area occupied by PD-L1 positive immune cells divided by tumor area x 100%
Tumor cell (TC) score	Area occupied by PD-L1 positive tumor cells divided by tumor area x 100%
TCIC score	Area occupied by PD-L1 positive tumor and immune cells divided by tumor area x 100%

**Table A2:** Therapies targeting the tumor microenvironment.

Target	Therapeutic Effect
**Immune Cells**	
Programmed cell death/programmed cell death-ligand 1 inhibitors	Restores cytotoxic T-cell response to cancer cells
Cytotoxic T-lymphocyte antigen-4 inhibitors	
Lymphocyte activation gene-3	Potential checkpoint targets under development
T-cell immunoglobulin and mucin domain containing protein-3	
T-cell immunoglobulin and ITIM domain	
B7-H3	
Chimeric antigen receptor T-cell therapy	Genetically engineered anti-tumor T-cells recognizing tumor antigens
**Cancer associated fibroblasts**	
Pegylated recombinant human hyaluronidase	Degrades intratumoral extracellular matrix
Matrix metalloproteinases inhibitors	Inhibitors extracellular matrix remodeling enzymes
Lysyl oxidase inhibitors	
Anti-fibroblast activation protein monoclonal antibodies	Monoclonal antibodies against surface biomarker of cancer associated fibroblasts
**Hypoxia**	
Vascular endothelial growth factor inhibitors	Inhibits tumor angiogenesis
Metastatic microenvironment	
RANK ligand inhibitor	Inhibits osteoclast function delaying skeletal related events
